# The Role of [^68^Ga]PSMA PET/CT for Clinical Suspicion of Prostate Cancer in Patients with or without Previous Negative Biopsy: A Systematic Review

**DOI:** 10.3390/cancers14205036

**Published:** 2022-10-14

**Authors:** Matteo Caracciolo, Angelo Castello, Luca Urso, Francesca Borgia, Naima Ortolan, Licia Uccelli, Corrado Cittanti, Massimo Castellani, Mirco Bartolomei, Massimo Lazzeri, Egesta Lopci

**Affiliations:** 1 Nuclear Medicine Unit, Oncological Medical and Specialists Department, University Hospital of Ferrara, 44124 Ferrara, Italy; 2 Nuclear Medicine Unit, Fondazione IRCCS Ca’ Granda, Ospedale Maggiore Policlinico, 20122 Milan, Italy; 3 Department of Translational Medicine, University of Ferrara, 44121 Ferrara, Italy; 4 Department of Urology, IRCCS Humanitas Research Hospital, Via Manzoni 56, 20089 Rozzano, Italy; 5 Nuclear Medicine Unit, IRCCS Humanitas Research Hospital, Via Manzoni 56, 20089 Rozzano, Italy

**Keywords:** 68Ga-prostate-specific membrane antigen, PSMA, PET/CT, mpMRI, prostate cancer, primary diagnosis, clinically significant cancer

## Abstract

**Simple Summary:**

In this paper we systematically evaluate the evidence regarding the role of [^68^Ga]PSMA PET/CT for clinical suspicions of prostate cancer in patients with or without previous negative biopsy. A critical review of PubMed and Web of Science according to the PRISMA statement was conducted. Eighteen publications were selected for inclusion in the analysis. In 8 articles, there was a direct comparison with mpMRI. [^68^Ga]PSMA PET/CT resulted more accurate in identifying primary prostate cancer with PSA values between 4 and 20 ng/mL than mpMRI. Moreover, its use combined with MRI improved sensitivity for csPCa detection, thus potentially avoiding unnecessary biopsies. Overall, [^68^Ga]PSMA PET/CT resulted a promising technique in patients with clinical suspicion of PCa and precedent negative biopsy or contraindications to MRI.

**Abstract:**

The purpose of the study is to systematically evaluate the evidence regarding the role of [^68^Ga]PSMA PET/CT for clinical suspicions of prostate cancer in patients with or without previous negative biopsy. We performed a critical review of PubMed and Web of Science according to the PRISMA statement. Eighteen publications were selected for inclusion in this analysis. QUADAS-2 evaluation was adopted for quality analyses. [^68^Ga]PSMA-11 was the radiotracer of choice in 15 studies, while [^68^Ga]PSMA-617 was used in another 3. In 8 articles, there was a direct comparison with mpMRI. The total number of patients included was 1379, ranging from 15 to 291, with a median age of 64 years (range: 42–90). The median baseline PSA value was 12.9 ng/mL, ranging from 0.85 to 4156 ng/mL. Some studies evaluated the PSMA uptake comparing the SUVmax of suspicious lesions with the SUVmax of the normal biodistribution to find out optimal cut-off points. In addition, some studies suggested a significant association between PSA levels, PSA density, and [^68^Ga]PSMA PET/CT finding. [^68^Ga]PSMA PET/CT seems to be more accurate in identifying primary prostate cancer with PSA values between 4 and 20 ng/mL than mpMRI. Moreover, in some trials, the combination of PSMA PET/CT and MRI improved the NPV in the detection of clinically significant prostate cancer (csPCa) than MRI alone. Our findings are limited by the small numbers of studies and patient heterogeneity. [^68^Ga]PSMA PET/CT is a promising technique in patients with clinical suspicion of PCa and precedent negative biopsy or contraindications to MRI. Furthermore, its use combined with MRI improves sensitivity for csPCa detection and can avoid unnecessary biopsies.

## 1. Introduction

Prostate cancer (PCa) is the second most common diagnosed malignancy and the fifth leading cause of cancer death among men worldwide [1]. According to the Gleason score (GS), PCa can be divided into clinically nonsignificant PCa (non-csPCa, GS = 6) and clinically significant PCa (csPCa, GS = 7–10). A prostate-specific antigen (PSA) blood sample twice per year and a digital rectal exam (DRE) once a year represent the only recommendations for patients affected by non-csPCa, as they have demonstrated long survival rates [2]. On the contrary, the diagnosis of csPCa is a primary need; therefore, early and accurate identification is fundamental in order to select the best therapy and improve clinical outcomes for these patients [3]. In this setting, very recently, the European Health Union proposed a new approach on cancer detection, introducing the proposal of a regular systematic monitoring of screening programs in order to cover in the future more target groups and more neoplasms; in particular for PCa, the recommendation is testing in men up to 70 on the basis of PSA level and magnetic resonance imaging (MRI) scanning as follow-up [4]. Traditionally, PCa screening for patients at risk is based on DRE, serum PSA testing, and eventually, histological examination by transrectal ultrasound (TRUS)-guided biopsy for definitive diagnosis. Furthermore, as strongly recommended by the European Association of Urology (EAU) guidelines on PCa active surveillance, in case of PSA progression or change in DRE or MRI findings, it is not suggested to progress to active treatment without a repeat biopsy [5,6]. Nevertheless, approximately 20% of patients undergo unnecessary biopsies due to a false-positive PSA test [7]. To overcome this issue, in the last years, multiparametric magnetic resonance imaging (mpMRI) has been introduced in the diagnostic workup of PCa. Indeed, high-spatial-resolution mpMRI images well correlate with histopathological PCa features [8]. Several studies have demonstrated that mpMRI is superior in terms of diagnostic accuracy with respect to transrectal template biopsy, reducing false-negative results [9,10]. Consequently, the recently updated guidelines of 2022 from the EAU mentioned above recommend mpMRI in biopsy-naïve patients, limiting systematic biopsies to lesions with a prostate imaging reporting and data system (PI-RADS) score greater than 3. The PRECISION trial reported that mpMRI can increase the detection rate of csPCa from 26% to 38% by reducing from 22% to 9% the identification of clinically nonsignificant PCa [11]. However, although the negative predictive value (NPV) is high, mpMRI still misses csPCa in up to 13% of cases with a rate of false positive up to 60%–80% also in lesions with a PI-RADS score higher than 4 [12,13,14]. Furthermore, MRI image analysis is based mainly on the PI-RADS 2.0, depending on the operator’s experience, leading to not negligible inter-reader variability [15]. In this setting, a new and accurate diagnostic technique with higher specificity was recently proposed. Prostate-specific membrane antigen (PSMA) is a membrane glycoprotein, with its typical intracellular, transmembrane, and extracellular domains. While in benign conditions PSMA is expressed in the cytoplasm, it migrates to the membrane side of malignant cells. Indeed, PSMA is overexpressed up to 1000 times in cancer cells more than normal prostate cells, and its level is increased in parallel with the tumor dedifferentiation, with a pathological overexpression also in several other malignancies, such as kidney cancer, bladder cancer, or hepatocellular carcinoma [16,17]. In the last few years, molecular imaging with PSMA positron emission tomography/computed tomography (PET/CT) has emerged as a useful tool complementing mpMRI’s role in the initial staging of PCa, along its proven capability to better display recurrent and metastatic lesions [18]. Our aim was to systemically investigate the diagnostic accuracy of PSMA PET/CT for the initial diagnosis of PCa in patients with clinical suspicion of malignancy or previous negative biopsy.

## 2. Materials and Methods

Preferred reporting items of the systematic review and meta-analysis (PRISMA) guidelines were used to conduct the present review [19].

### 2.1. Literature Search Strategy and Study Selection

An independent systematic search was conducted in PubMed and Web of Science for articles published up to 28 February 2022. The search keywords were as follow: ((((Prostat* AND (Cancer OR malignan* OR adeno- carcinoma OR lesion* OR Disease)) AND (PSMA OR “prostate-specific membrane antigen positron emission tomography”)) AND (MR OR magnetic resonance imaging OR MP-MRI OR multi-parametric MRI OR multi- parametric magnetic resonance imaging OR multipara- metric MRI OR “multiparametric magnetic resonance imaging”)) AND Diagnosis) combined with ((((Prostat* AND (Cancer OR malignan* OR adeno- carcinoma OR lesion* OR Disease)) AND ((PSMA OR “prostate-specific membrane antigen positron emission tomography”)) AND (PET OR “positron emission tomography”) AND (gallium OR ga)) AND Diagnosis AND Biopsy). Two authors (M.C. and A.C.) performed additionally a search through the references of the articles, through unpublished and ongoing studies in the ClinicalTrials.gov database. After initial screening by title and abstract text, the subset of potentially eligible articles was selected as full texts. Disagreements were analyzed by a third author (E.L.).

The studies included in the analysis reported on the diagnostic accuracy of PSMA PET/CT for the primary detection of PCa in patients with clinical suspicion. Suspicion of PCa was defined by urinary symptoms, elevated PSA value, and abnormal DRE or TRUS findings. We included both studies investigating biopsy-naïve patients and studies of cases prior to negative biopsy results. On the other hand, we excluded studies with PCa already proven by biopsy, studies of a radiotracer other than [^68^Ga]PSMA, and those analyzing only the accuracy of PET/MRI. The exclusion criteria were as follows: (a) non-English articles; (b) studies in animal models; and (c) case reports, poster presentations/conference abstracts, and letters to editor.

### 2.2. Data Extraction

All data were selected based on standardized criteria and were independently checked by the reviewers. The following data from the included studies were extracted: author, publication year, study design, number of patients included, median age, median PSA value, agent and activity administered, and criteria for positive scan. Sensitivity, specificity, PPV, NPV, and accuracy for the evaluable articles were estimated, evaluating the diagnostic accuracy of PET/CT in the detection of csPCa. When included in the article, a comparison with mpMRI was also evaluated.

### 2.3. Quality Assessment

The QUADAS-2 tool was adopted to evaluate the risk of bias. This tool is based on a score analysis of four domains: patient selection, index test, reference standard, and flow and timing. The bias evaluation was conducted to assess the applicability and reliability of the data produced [20,21].

## 3. Results

### 3.1. Literature Search

**Figure 1:**
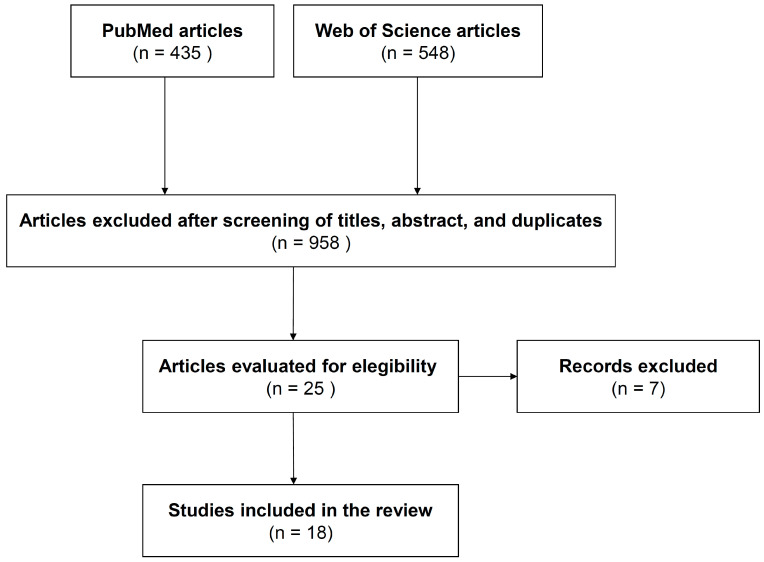
Flowchart of the study following PRISMA guidelines.

Based on the systematic search, 435 articles were found by PubMed and 548 by Web of Science. After initial screening according to titles, eligible criteria, and removal of duplicates, a total of 25 articles were selected. Of these, 18 were finally included and downloaded in a full-text version [22,23,24,25,26,27,28,29,30,31,32,33,34,35,36,37,38,39]. No further studies were added after examination of the references of these articles (Figure 1: PRISMA flowchart of the study).

### 3.2. Basic Characteristics

The studies included in our review were published at an interval time between 2017 and 2022. Twelve studies (66.6%) enrolled more than 50 patients, and 4 (22.2%) more than 100; 9 studies (50%) were retrospective, and the other 9 (50%) were prospective. Sixteen out of 18 studies (88.9%) were conducted in a single center, while only 2 (11.1%) in more than 1 center. The total number of patients included was 1379, ranging from 15 to 291, with a median age of 64 years (range: 42–90) (Table 1: Baseline characteristics).

### 3.3. Imaging and Technical Aspects

[^68^Ga]PSMA-11 PET/CT scans were performed in 15 articles, while [^68^Ga]PSMA-617 was used only in 3. Acquisition time was started after 60 min from radiotracer administration in 16 studies, while in 2 studies, another delayed scan of the pelvis after 20 mg injection of furosemide at 120 min was performed. PET/CT scans were evaluated visually and semiquantitatively. Sixteen out of 18 articles had specified criteria for imaging evaluation: 12 (66.7%) with focal uptake more than background, 3 with focal uptake more than liver, and only 1 with SUVmax > 2.5. Two experienced nuclear medicine specialists always interpreted results by consensus. In 8 articles, there was a direct comparison with MRI [22,23,26,28,31,32,33,35]. In 7 of them, MRI scans were acquired on 3.0 Tesla (T) MRI by four sequences: T1-weighted imaging, T2-weighted imaging, fat-suppression presaturation-attenuated inversion recovery, and diffusion-weighted imaging (DWI); PI-RADS v2.0 was used for reporting images in all of them. Only 1 study used a 1.5 T MRI. Total prostatectomy or prostate biopsy defining csPCa as Gleason score ≥ 7 or based on the International Society of Urological Pathology (ISUP) grade system represented the standard reference. In particular, 12 out of 18 articles used TRUS-guided biopsy as gold standard [22,23,24,25,26,29,31,33,35,36,37,39], 4 PET/TRUS fusion–guided prostate biopsy [27,30,32,38], and 2 radical prostatectomy specimen [28,34].

### 3.4. Main Findings

In the included studies, no significant adverse events were reported after the administration of either [^68^Ga]PSMA-617 or [^68^Ga]PSMA-11. Overall, [^68^Ga]PSMA uptake was positively correlated with PSA levels and GS, suggesting PET/CT as a useful tool in the diagnosis of PCa. Furthermore, the higher tumor differentiation, the higher [^68^Ga]PSMA uptake, with SUVmax of tumors in high-risk PCa subjects more elevated than those in intermediate-risk PCa patients.

Some papers evaluated PSMA uptake, comparing the SUVmax of suspicious lesions with the SUVmax of normal biodistribution [27,31,32,38]. Lopci et al. [38] showed that a SUVmax > 4.8 discriminated malignant from benign lesions with a sensitivity and specificity of 82.4% and 72.2% (AUC 0.843), respectively. When considering only csPCa (i.e., GS ≥ 7) compared with all other lesions (i.e., benign and those with GS = 6), SUVmax and SUVratio showed optimal cut-off points of 5.4 and 2, respectively, with a sensitivity of 100% for both and specificities of 76% and 88%, respectively. Furthermore, Chen Liu et al. [27] underlined that the median highest SUVmax in prostate lesions was 5.61 (2.90–30.95), while the median background SUVmax in the prostate gland was 3.40 (2.00–4.40); according to univariate logistic regression analysis, the SUVmax of cancer lesion, tumor-to-normal-prostate background SUVmax, and tumor-to-normal-liver background SUVmax were potential predictors for csPCa in patients undergoing repeated biopsy. SUVmax > 4.795 and SUVratio > 2.040 were able to distinguish malignant lesions from benign pathologies in a study conducted by Yu Li and colleagues [31]^.^ In addition, when lesions were divided into nonclinically significant PCa versus csPCa, the cut-off values for SUVmax and for SUVratio were 5.050 and 2.365, respectively. Moreover, the same Italian group mentioned above highlighted that for suspected prostate lesions, the median values for SUVmax, background SUV, and SUVratio were 4.29 (1.48–50.66), 2.48 (1.36–4.35), and 1.67 (1–16.66), respectively. In comparison with pathology, the median SUVmax and SUVratio were significantly higher for malignant lesions than those of benign nature, as well as for GS ≥ 7 compared with either a GS of 6 or benign conditions (*p* < 0.001). SUVmax > 5.4 and SUVratio > 2.2 well identified csPCa with accuracies of 81% and 90%, respectively [30].

In addition, some studies suggested an association among PSA levels, PSA density, and [^68^Ga]PSMA PET/CT finding. For example, in a study by Chandra and colleagues [33], the PCa group showed higher values of mean PSA, PSA density, and prostate-to-liver SUVmax ratio compared with the benign group. In the same work, patients with PSA greater than 20 ng/mL had higher SUVmax than those with PSA below 20 ng/mL (19.1 ± 20.6 vs. 6.01 ± 5.4, *p* = 0.0052). Likewise, SUVmax was higher in patients with PSA density > 0.15 than those with PSA density ≤ 0.15 (11.8 ± 15.4 vs. 5.4 ± 2.89, *p* = 0.038). Moreover, Le-Le Zhang et al. [29] recruited and randomized into two groups (i.e., [^68^Ga]PSMA-11 PET/CT and TRUS) a total of 120 patients with PSA levels > 4 ng/mL for the diagnosis of PCa, in particular, csPCa. In the group that performed [^68^Ga]PSMA PET/CT, PCa was detected in 26/60 (43.3%) patients, and csPCa in 24/60 (40%). On the other hand, in the TRUS group, PCa was detected in 19/860 (31.6%) patients and csPCa in 15/60 (25%) patients. Moreover, among patients within the [^68^Ga]PSMA PET/CT group, the percentage of PCa and csPCa was significantly higher in patients with positive PET/CT scan than those with PET/CT negative scan (23/25, 92% vs. 3/35, 8.6%, χ^2^ = 41.34, *p* < 0.01 for PCa, and 22/25, 88% vs. 2/35, 5.7%, χ^2^ = 42.68, *p* < 0.01 for csPCa). Among patients with PSA comprising between 4.0 and 20.0 ng/mL, PSMA PET/CT identified more csPCa than the TRUS-guided biopsy group (10/37, 27.0% vs. 3/34, 8.8%, χ^2^ = 3.93, *p* < 0.05), suggesting [^68^Ga]PSMA PET/CT as a better alternative in patients with low PSA levels (<20.0 ng/mL) and improving the detection rate of csPCa.

Moreover, Zhang J. et al. [36] evaluated the diagnostic performance of [^68^Ga]PSMA PET/CT in the detection of suspected PCa in patients with PSA from 0.4 to 50 ng/mL, comparing PET/CT efficacy with that derived from the European Randomized Study of Screening for Prostate Cancer Risk Calculator Version 3 (ERSPC-RC3) and Prostate Cancer Prevention Trial Risk Calculator (PCPT-RC) nomograms. Among 58 histological samples, [^68^Ga]PSMA PET/CT would have avoided 11 (19%) unnecessary biopsies, as suggested instead by *ERSPC-RC3.*

To the best of our knowledge, Kumar et al. [35] compared the diagnostic accuracy of mpMRI vs. PET/CT for the first time. The study included 15 patients with a serum PSA ranging from 4 to 20 ng/mL. MRI was unable to identify tumor lesions in 8 out of 15 patients (53.3%) and was suggestive of malignancy (PI-RADS > 2) in the other 7 patients, whereas PI-RADS 3 was the most commonly reported score. On the contrary, [^68^Ga]PSMA PET/CT identified focal lesions of increased uptake in 10 out of 15 patients (66.7%) with a mean SUVmax of 13.8 (range, 4.5–23). Thus, the combination of MRI and [^68^Ga]PSMA PET/CT findings improved lesion detection in 11 of 15 patients (73.3%). Afterwards, all patients underwent standard 12-core TRUS-guided biopsy, which was diagnostic of malignancy in 9 out of 15 patients (60%). The sensitivity, specificity, PPV, NPV, and diagnostic accuracy for mpMRI were 62.5%, 71.4%, 62.55, 71.4%, and 66.6%, respectively. For [^68^Ga]PSMA, PET/CT sensitivity, specificity, PPV, NPV, and diagnostic accuracy were 88.8%, 66.65%, 80%, 80%, and 80%, respectively. Comparing a histopathological report with imaging, two false-positive and three false-negative patients were identified by MRI. Similarly, two false-positive and one false-negative patients were diagnosed by [^68^Ga]PSMA PET/CT. Moreover, according to [^68^Ga]PSMA PET/CT findings, biopsy could be avoided in 4 (26.6%) patients. Similarly, Chandra et al. [33] reported 10 discordant cases in a cohort of 65 patients; of note, all these discordant cases derived from positive MRI (PI-RADS 4–5) and negative PSMA PET/CT (miPSMA-ES < 2), with 7 out of 10 patients coming out negative in biopsy.

**Figure 2:**
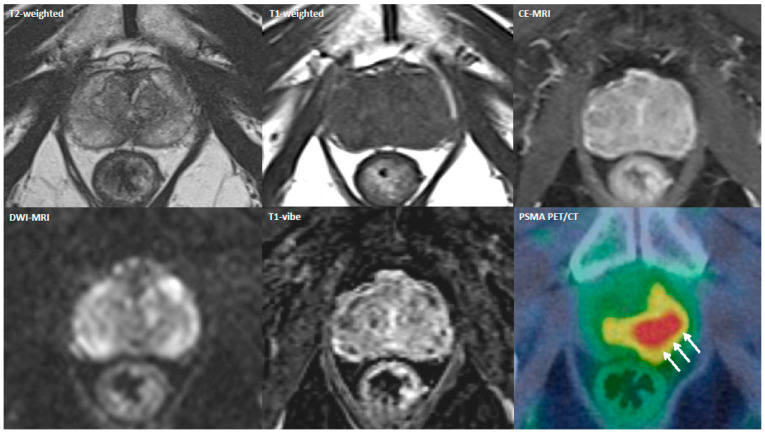
Multipanel comparison between mpMRI (PI-RADS 3) and [^68^Ga]PSMA PET/CT (SUVmax 4.3; SUVratio to background 2.1) in a biopsy-naïve patient with PSA of 5.54 ng/mL. The highlighted lesion resulted in a GS 3+4 adenocarcinoma of the left prostatic lobe (white arrows). PSMA = prostate-specific membrane antigen; PET/CT = positron emission tomography/computed tomography; CE − MRI = contrast − enhanced MRI; DWI − MRI = diffusion − weighted imaging MRI.

However, Lopci and colleagues evaluated the diagnostic accuracy of [^68^Ga]PSMA PET/CT in a group of 97 patients who had persistently elevated PSA, negative rectal examination, previous negative biopsy, and/or previous negative MRI scan. PET/CT identified csPCa in 4 patients who had a previous negative MRI and also in 7 patients with previous positive MRI and negative biopsy. Considering PI-RADS ≥ 3, mpMRI demonstrated a diagnostic accuracy of 48% with a sensitivity, specificity, PPV, and NPV of 81%, 26%, 41%, and 68%, respectively; diagnostic accuracy was slightly increased to 57% for PI-RADS ≥ 4; on the other hand, PSMA PET/CT showed an accuracy, sensitivity, specificity, PPV, and NPV of 84%, 60%, 97%, 92%, and 81%, respectively [38]. In another direct comparison by Yu Li et al. [31], [^68^Ga]PSMA PET/CT came out more accurate than mpMRI in the primary diagnosis of PCa in patients with PSA levels comprising between 4 and 20 ng/mL (Figure 2: Comparative example). In particular, PSMA PET/CT showed higher AUC than mpMRI (0.881 vs. 0.689, *p* = 0.0019), suggesting a potential better diagnostic performance of PET/CT.

Four studies investigated whether the combination of PET/CT and MRI is superior to the two methods alone. A retrospective study by Liwei Wang et al. [28] evaluated the combination of apparent diffusion coefficient (ADC) from MRI with SUVmax on [^68^Ga]PSMA PET/CT for the differentiation of PCa from benign lesions. MRI missed five malignant prostatic lesions, which were detected instead by [^68^Ga]PSMA PET/CT, showing high PSMA avidity but no significant modifications on ADC maps. The ADC value negatively correlated with SUVmax (*p* = 1.52 × 10^−9^ in all lesions, *p* = 4.54 × 10^−2^ in benign lesions, and *p* = 7.94 × 10^−3^ in PCa). Sensitivity and specificity were 67.2% and 97.7%, respectively, when the SUVmax cut-off value was 11.72 (Youden index = 0.65, AUC = 0.905). The combination of [^68^Ga]PSMA PET/CT and MRI, with a cut-off value for SUVmax/ADC of 12.35, determined a sensitivity and specificity of 81.2% and 88.4%, respectively, suggesting SUVmax/ADC as a potential predictor for PCa. Similar results were obtained by Nuo et al. [22] in a retrospective study including 105 patients with suspected PCa. The sensitivity and specificity of MRI combined with PET/CT for the diagnosis of PCa were 94% and 81%, respectively (Youden index and AUC of 0.75 and 0.90, respectively). Furthermore, the Youden index and AUC were significantly higher than values when considering MRI or PET/CT alone. 

In support of these data, the results of the first multicenter prospective trial (PRIMARY) for the diagnosis of PCa in men with PI-RADS 2–5 were recently disclosed. Combined PSMA PET/CT + MRI improved NPV for detecting csPCa compared with MRI alone (91% vs. 72%, *p* < 0.001); the combination also increased sensitivity (97% vs. 83%, *p* < 0.001), allowing to safely omit biopsy in a further proportion of men [23].

### 3.5. Assessment for the Risk of Bias

The QUADAS-2 tool was used to assess the risk of bias (Table 2: QUADAS-2 score). Nine out of 18 studies recruited patients prospectively, but all the studies included only patients with clinical suspicion of PCa or previous negative biopsy without a proven histopathology diagnosis. It should be noted that in 8 studies, the results of the reference standard were interpreted knowing the results of PET/CT or mpMRI scan to evaluate their accuracy [22,24,27,29,32,34,35,38]. For applicability concerns, low concern for patient selection emerged for all studies, while 2 articles showed high concern about the index test [29,39] and 3 for the reference standard [23,29,31].

## 4. Discussion

Prostate cancer causes more than 360,000 deaths a year worldwide and an ever-increasing incidence [40]. TRUS-guided biopsy is a standardized technique for the diagnosis of PCa; nevertheless, the percentage of false negative is still high, ranging from 15% to 35% [41]. Due to the high image quality and resolution provided by MRI, the detection rate of csPCa has increased, allowing for avoiding unnecessary biopsies [42]. However, some studies have shown that there is a high rate of false positives (i.e., prostatitis) confirmed by histopathological data and/or a significant loss of some prostatic and extraprostatic lesions [43]. Indeed, it has been demonstrated that mpMRI missed and underestimated approximately 5%–15% of csPCa lesions, in particular, those with a diameter below 1 cm and those located in the central and transitional zones, which limits the diagnostic power of mpMRI [44]. Moreover, several cases of PCa are slow growing and asymptomatic, making early diagnosis difficult; consequently, finding accurate diagnostic methods for the detection of csPCa is mandatory to improve the survival rate and to set up the correct management of these patients [45]. [^68^Ga]PSMA PET/CT is a noninvasive, easy-to-perform technique that can be used in patients with absolute or relative contraindications to MRI. It provides diagnostic imaging and can detect distant metastasis in the same scan, thus impacting a patient’s prognosis [46]. Furthermore, since biopsy sometimes may not be reliable or some patients might refuse it, PET/CT can be helpful in cases still highly suspicious for PCa. Despite that, studies evaluating the role of [^68^Ga]PSMA PET/CT in cases with suspected PCa are few. Therefore, in our systematic review, we tried to summarize published data in the literature in order to analyze more accurately the potential application of [^68^Ga]PSMA PET/CT in this context [47]. 

In the studies herein analyzed, a significant correlation between the intensity of [^68^Ga]PSMA uptake and PSA level/GS in the confirmed lesions was found. Furthermore, SUVmax and SUVratio of the primary tumor could be used as predictors of csPCa [27,31,32,38]. Recently, in their meta-analysis, Satapathy et al. [48] investigated the role of [^68^Ga]PSMA PET/CT for the initial diagnosis of PCa based on clinical or biochemical suspicion. The pooled sensitivity and specificity were 0.97 (95% confidence interval (CI): 0.90–0.99) and 0.66 (95% CI: 0.52–0.78), respectively. The pooled positive and negative likelihood ratios were 2.86 (95% CI: 1.95–4.20) and 0.05 (95% CI: 0.01–0.15), respectively. Finally, the AUC of summed ROC curves was 0.91 (95% CI: 0.88–0.93). In a more recent meta-analysis by Zhao et al. [49], PSMA PET/CT outperformed MRI for detecting primary PCa, with a pooled sensitivity of 0.93 vs. 0.87 (*p* < 0.01). On the other hand, both techniques were similar for the localization of the lesions.

Previously, some studies have shown that [^68^Ga]PSMA PET/CT could be a valid alternative or addition in patients with suspected primary PCa or precedent negative MRI scan. In recent years, Simopoulus et al. [50] reported the first case of a successful [^68^Ga]PSMA PET/CT and MRI/ultrasound-guided biopsy; the PSMA-positive region of interest was transposed onto biplanar MRI images, and a target biopsy was performed, demonstrating a GS 3+4 PCa. Subsequently, Lopci et al. [38] confirmed PET/CT usefulness in a cohort of 45 patients with a negative prostate biopsy and negative/uncertain MRI images; all patients underwent PET/CT scan and received PET/ultrasound-guided target biopsy with a detection rate of PCa of 44%. Therefore, [^68^Ga]PSMA PET/CT’s capacity to detect PCa in cases with negative findings at MRI could implement the imaging landscape for selected patients [51]. Recently, the results of the PRIMARY trial proved that through the combination PSMA PET/CT and MRI, the sensitivity and NPV for csPCa improves compared with MRI alone, allowing for avoiding unnecessary biopsies in men with negative PSMA + MRI findings [23]. Therefore, the optimal imaging approach would probably be the combination of the two techniques, given the advantage of defining csPCa in particular in case of uncertain results (i.e., PI-RADS 3 lesions) [52]. However, except the trial above cited, most studies comparing [^68^Ga]PSMA PET/CT and MRI are retrospective and are conducted in patients with already-diagnosed PCa [53,54,55]. Indeed, there is an urgent need for new prospective studies investigating the role of PET/CT in the detection of PCa. The number of ongoing clinical trials is 42, but only 8 considered patients without a diagnosis of PCa, and to the best of our knowledge, there is only 1 recruiting trial comparing the diagnostic performance of the two modalities. Our group recently developed a dedicated trial comparing mpMRI and [^68^Ga]PSMA-PET/CT (ClinicalTrials.gov Identifier: NCT05297162). This is a prospective single-arm case-control study aimed to evaluate in parallel PSMA PET/TRUS with mpMRI/TRUS fusion prostate biopsy in patients at high risk of PCa after at least one negative biopsy (PROSPET-BX) (Table 3: Ongoing trials). 

However, [^68^Ga]PSMA PET/CT has some drawbacks, such as the need for an on-site generator to produce the nuclide, which has a short half-life, and a prevailing urinary excretion leading to increased PET artifacts surrounding the bladder. In this regard, a recent add-on is represented by fluorine-18 [^18^F]-labelled PSMA-1007 that has a longer half-life and an improved image resolution [56,57]. In addition, despite that PSMA PET/CT-TRUS fusion-assisted targeted biopsy has a high detection rate for csPCa and allows a real-time visualization for target lesions, it is not free from some difficulties, for example, the expertise of the operator in the analysis and reporting of the findings or fusion errors of the dedicated software derived from the degree of bladder distension, different body positions, and the pressure of the probe [58].

Additionally, as biopsy is performed using a single-puncture technique and focused on the most PSMA-avid lesion, tumors with negative or low PSMA expression might be missed by PSMA PET, although this circumstance represents only a small percentage of prostate cancer at primary diagnosis (i.e., 5%) [59,60].

Moreover, in a recent study by Zeng et al. [61], the diagnostic value of [^18^F]PSMA-1007 PET/MRI for the detection of PCa was evaluated and compared with MRI alone: similar sensitivities were reported for [^18^F]PSMA-1007 PET/MRI and MRI, while the specificity, PPV, and NPV of [^18^F]PSMA-1007 PET/MRI were superior than those of MRI alone, with values of 100%, 100%, 83.33%, and 87.5% vs. 50%, 87.50%, and 62.5%, respectively. Moreover, in a review by Evangelista et al. [62], the performance of PET/MRI in primary diagnosis was reported, with a pooled sensitivity and specificity for the per-patient and per-lesion of 61.5% and 90.9% and 94.9% and 62.5%, respectively. Indeed, the principal advantages of PET/MRI are lower radiation dose to the patient and higher soft-tissue contrast, but some limitations remain with regard to the high costs and the validation of quantitative uptake metrics [63]. Nevertheless, the availability of this tool is currently low, and other studies are needed to evaluate its real efficacy compared with mpMRI and PSMA PET/CT alone. 

Another aspect that should be considered before the introduction of PSMA PET/CT into clinical routine is its impact in terms of costs and outcomes. A recent cost-effectiveness analysis, based on information from a proPSMA trial, documented costs of approximately AUD 1000 and AUD 1400 saved per additional accurate detection of nodal disease and distant metastases, respectively [64]. Despite that these results were derived from Australian health, they might be translated also for European countries as gallium-68 generators are used to produce the PSMA radiotracer. Recently, an Australian group evaluated the cost efficacy between [^68^Ga]PSMA-11 and [^18^F]PSMA-1007 for the detection of recurrent PCa. Although data showed a slightly higher detection rate for [^18^F]PSMA-1007, these were not reflected into a better clinical performance or into a higher cost efficacy [65]. When it comes to MRI, a recent systematic review showed that the use of MRI before TRUS-guided biopsy is more cost-effective than TRUS alone. The results suggest that the use of MRI-US fusion targeted biopsy in the initial diagnosis of PCa can be a potential cost-effective strategy [66].

Some limitations of the current review need to be pointed out. First, the number of studies considered was small, with the majority deriving from a single center and being retrospective, with possible selection bias. Farther, study design and patient heterogeneity are additional limitations. Because of this heterogeneity, we did not perform a meta-analysis on the diagnostic accuracy of [^68^Ga]PSMA PET/CT for the detection of PCa considering that the differences among included studies would have caused a significant source of bias [67]. 

To summarize, [^68^Ga]PSMA PET/CT has shown good performance in the initial diagnosis of PCa in patients with clinical and/or laboratory suspicions for PCa. Evidence in the last decade is accumulating, supporting high concordance between PSMA PET/CT and histopathology for csPCa identification, which is at least equal, if not even superior, to mpMRI. These aspects open an interesting and promising insight for the future of the PSMA-based imaging and targeted biopsy. Moreover, its use combined with MRI improves the sensitivity for csPCa and can avoid unnecessary biopsies. Further randomized and larger prospective studies comparing the two methods in this setting are needed.

## Figures and Tables

**Table 1:** Summary of patients’ characteristics and diagnostic performance of different imaging modalities, including PSMA PET/CT.

Author	Year	Patients	Study Design	Age (Median)	PSA (ng/mL)	Imaging	Sensitivity	Specificity
Hoffmann [39]	2017	25	Retrospective	67	20.4 ± 33.5 mean	SUVmax 5.4	94%	100%
Lopci [38]	2018	45	Prospective	64	10.46 mean	SUVmax 5.4	100%	76%
						SUVratio 2	100%	88%
Sasikumar [37]	2018	118	Prospective	67	11.56 (0.85–4156) median	NR	NR	NR
Zhang J [36]	2019	58	Retrospective	70	19.46 (1.31–49.07) median	PET/CT	91.67%	81.82%
Kumar [35]	2019	15	Prospective	66.2	9.9 (5.1–19.5) mean	PET/CT	88.8%	66.65%
						MRI	62.5%	76.4%
Chen [34]	2019	54	Retrospective	65	13.53 (4.04–110) median	PET/CT	89%	71%
						MRI	76%	89%
Chandra [33]	2020	64	Retrospective	70	15.67 (1.74–44) mean	PET/CT	74%	92%
						SUVmax 5.6	95%	90.9%
Lopci [32]	2020	97	Prospective	74.7	7.6 (1.86–32.6) median	SUVmax 5.4/SUVratio 2.2	60%	97%
						MRI	81%	26%
Li [31]	2020	67	Retrospective	68	10.48 (3.15–19.76) mean	PET/CT	87.9%	88.2%
						MRI	84.9%	52.9%
Lopci [30]	2020	20	Prospective	74.7	7.6 (1.86–32.6) median	SUVmax 5.4	60%	93%
						SUVratio 2.2	80%	93%
Zhang L [29]	2020	120	Prospective	71.1	28.2 ± 26 mean	PET/CT vs. TRUS	NR	NR
Wang [28]	2020	63	Retrospective	69.56	4.15–1298 mean	ADC 1.02 × 10^−3^	58.1%	90.6%
						SUVmax 11.7	67.2%	97.7%
Liu [27]	2020	31	Prospective	65	18.0 (5.48–49.77) median	PET/CT	100%	68.4%
Chinnappan [26]	2021	67	Retrospective	70	23.2 (2.97–45.6) mean	PET/CT	76.3%	96.5%
						MRI	92.1%	65.5%
Jiao [25]	2021	58	Retrospective	70.6	40.84 (13.66–89.96) median	SUVmax 5.3	85.8%	86.2%
Jain [24]	2021	81	Prospective	68.4	10.5 ± 4.6 mean	SUVmax 6.15	84%	80%
Emmett [23]	2022	291	Prospective	64	5.6 (4.2–7.5) median	PET + MRI	97%	40%
						MRI	83%	53%
Nuo [22]	2017	105	Retrospective	68.4	3.45–1000 mean	SUVmax 12.9	74%	94%
						bpMRI	62%	88%
						bpMRI/PET	80%	88%

Abbreviations: ADC, apparent diffusion coefficient; bpMRI, biparametric magnetic resonance imaging; NR, not reported; PET/CT, positron emission tomography/computed tomography; TRUS, transrectal ultrasonography-guided biopsy; SE/SP, sensitivity/specificity.

**Table 2:** QUADAS-2 results showing the assessment for the risk of bias and risk of applicability for the studies that were included in the analysis.

Study	Risk of Bias				Applicability Concerns		
	Patient Selection	Index Test	Reference Standard	Flow and Timing	Patient Selection	Index Test	Reference Standard
Hoffmann [39]	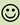	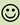	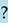	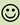	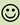	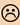	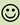
Lopci [38]	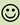	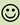	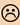	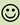	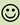	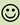	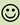
Sasikumar [37]	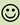	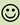	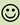	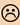	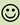	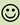	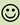
Zhang J [36]	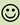	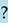	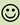	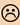	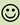	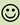	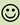
Kumar [35]	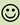	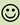	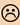	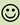	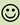	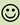	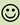
Chen [34]	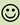	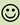	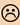	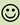	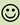	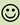	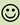
Chandra [33]	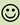	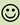	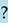	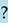	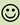	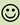	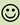
Lopci [32]	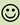	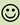	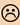	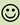	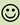	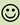	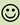
Li [31]	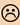	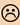	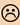	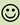	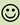	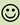	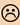
Lopci [30]	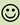	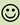	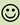	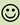	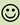	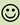	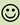
Zhang L [29]	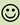	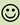	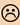	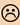	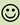	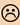	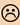
Wang [28]	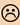	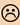	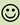	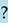	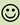	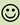	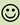
Liu [27]	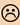	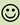	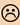	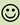	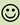	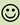	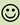
Chinnappan [26]	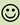	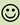	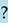	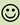	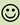	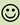	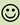
Jiao [25]	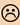	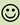	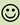	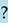	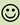	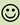	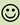
Jain [24]	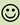	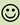	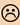	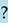	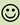	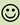	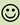
Emmett [23]	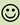	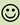	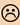	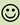	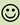	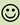	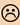
Nuo [22]	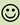	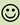	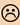	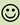	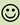	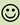	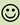

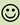
 Low risk; 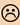 high risk; 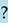 unclear risk.

**Table 3:** Summary of the ongoing clinical trials with PSMA PET/CT for the initial detection of PCa (source: https://clinicaltrials.gov/), accessed up to 28 February 2022.

Trial Identifier Number	Phase	Status	Radiotracer	AIM	Country
NCT05154162	III	Recruiting	Not specified	To evaluate PSMA PET/CT additive value for sPCa diagnosis in men with negative/equivocal MRI	Australia
NCT02282137	II	Recruiting	[^68^Ga]PSMA-11	Sensitivity of PSMA PET/CT for the detection of tumor location	USA
NCT04179968	NA	Recruiting	[^68^Ga]PSMA-11	To assess the ability of PSMA PET/CT to increase diagnostic accuracy in localizing primary and metastatic lesions in patients with suspected prostate cancer and elevated PI-RADS scores and PSA	USA
NCT05002465	NA	Available	[^68^Ga]PSMA-11	To provide PSMA PET/CT for clinical use in the diagnosis, staging, and restaging	USA
NCT05297162	NA	Recruiting	[^68^Ga]PSMA-11	To compare PSMA PET/TRUS vs. mpMRI/TRUS fusion prostate biopsy in men with a high suspicion of PCa after at least one negative biopsy	Italy
NCT05160597	I	Recruiting	[^68^Ga]PSMA-11	To identify prostate cancer in men with a prior negative or inconclusive prostate biopsy	USA
NCT05137561	NA	Recruiting	[^68^Ga]PSMA	To compare the diagnostic yield of robotic-arm-assisted PSMA PET/CT–guided prostate biopsy and MRI-directed TRUS-guided prostate biopsy in patients with PI-RADS grading of 4/5	India
NCT04867603	III	Recruiting	[^68^Ga]PSMA	Diagnostic performance of digital PET/CT using 68Ga PSMA for the characterization of prostate lesions	USA

Abbreviations: mpMRI, multiparametric magnetic resonance imaging; NA, not available; PI-RADS, prostate imaging reporting and data system; PSA, prostate-specific antigen; PSMA PET/CT, prostate-specific membrane antigen positron emission tomography/computed tomography; sPCa, significant prostate cancer; TRUS, transrectal ultrasonography-guided biopsy.
